# Evaluation of Soft Tissue Changes Following Orthognathic Surgery Using Virtual Surgical Planning Software

**DOI:** 10.7759/cureus.63495

**Published:** 2024-06-30

**Authors:** Abhirami Srikanth, Shanmugasundaram Somasundaram, Krishnakumar Raja

**Affiliations:** 1 Oral and Maxillofacial Surgery, Sri Ramaswamy Memorial (SRM) Dental College, Chennai, IND

**Keywords:** recent advances, post-operative changes, lefort 1 osteotomy, soft tissue changes, virtual surgical planning, bsso, orthognathic surgery

## Abstract

Context: Orthognathic surgery brings about a harmonious relationship between jaws, resulting in improved facial aesthetics. It is key to analyze if satisfactory results can be attained by using virtual surgical planning for orthognathic procedures so as to recommend it for routine clinical practice.

Aims: The aims of this study were to evaluate the various soft tissue changes that take place following orthognathic surgery using three-dimensional (3D) imaging and virtual surgical planning software and quantify the accuracy of virtual surgical planning software on patients undergoing orthognathic surgery.

Settings and design: This is an observational prospective study with a sample size of 12.

Methods and materials: In this prospective study, 12 patients undergoing orthognathic surgery were included following the inclusion and exclusion criteria. A usual pre-surgical work-up was done and a 3D replica of the facial skeleton was formulated using the software with the DICOM data acquired from the patient i.e., CT and scans of patient’s dentition. Virtual surgical planning was done and splints were manufactured according to the desired newly achieved position. Patients were operated following the virtual surgical plan guided by the computer-aided design and computer-aided manufacturing (CAD-CAM) splints. Post-operative evaluation was done. As the study is more of a descriptive study to obtain detailed knowledge of a new procedure, only one group is being studied and hence there is no statistical testing included in this study.

Results: The mean discrepancy noticed after superimposition of soft tissue points was 0.92 with a standard deviation of 0.3.

Conclusions: 3D CT virtual surgical planning is a reliable tool to achieve predictable and reliable post-operative results in orthognathic surgical cases.

## Introduction

Orthognathic surgery, considered an elective surgical procedure, brings about a harmonious relationship between the jaws, the maxilla, and the mandible, ideally leading to an enhanced facial profile and aesthetics. A balance between both hard tissues and soft tissues of the face is of utmost importance to attain the most effective post-operative outcome [1].

In this continually progressing digital era, advanced 3D visual aids and virtual surgical planning (VSP) tools play an important role in achieving maximal efficiency, aiding surgeons as well as patients toward faster treatment planning and lesser post-operative complications albeit with the best possible results. It also helps in avoiding human errors which usually occur during conventional treatment planning [2].

The vast technological strides seen in the field of Maxillofacial Surgery, with the advent of 3D printing, computer-aided design and computer-aided manufacturing (CAD-CAM) splint-guided surgeries, patient-specific implants, robotically assisted surgeries, etc., give orthognathic surgeons a wide scope and potential for better analysis of the craniofacial skeleton and improved result prediction.

Traditional surgical training is recurrently resisted by concerns of patient safety, the cost of operating rooms, and other complications. Virtual reality training allows a surgeon to achieve skills in less time before operating on any patient. The development of technology and hardware has significantly improved patient safety, increased surgical skills, and produced expert surgeons. It has been proven in Western countries that virtual reality is an effective solution for improving surgical skills, but the awareness and use of such kind of surgical skill training is questionable in the Indian scenario. Despite the numerous advantages offered by these tools, only a handful of maxillofacial surgeons use them in their routine clinical practice in India due to a lack of awareness of the technique involved [3].

This study’s main objective would be to evaluate the level of accuracy and precision achieved by three-dimensional (3D) CT planning using virtual surgical planning software, by analyzing the soft tissue changes that take place post-operatively and also to formulate a schematic workflow so as to adapt VSP in routine surgical practice.

## Materials and methods

Study design

This study is an observational prospective study including patients requiring orthognathic surgery for either functional or aesthetic improvement.

Patient selection

Patients reporting to the Department of Oral and Maxillofacial Surgery, SRM Dental College and Hospital, during the period of 2020 to 2022 were selected following the specific inclusion and exclusion criteria. Informed consent was obtained from all patients.

Inclusion Criteria

All patients diagnosed with dentofacial deformities undergoing orthognathic surgery willing to take part in the study and patients aged 18 to 30 years were included.

Exclusion Criteria

Patients not willing to take part in the study and long-term follow-up, syndromic patients, cleft patients, patients contraindicated for CT, and medically compromised patients were excluded.

The sample size was calculated based on the study by Julious (sample size of 12 per group rule of thumb for a pilot study) [4], and 12 operated cases were selected and included in the study.

Study methodology

**Figure 1:**
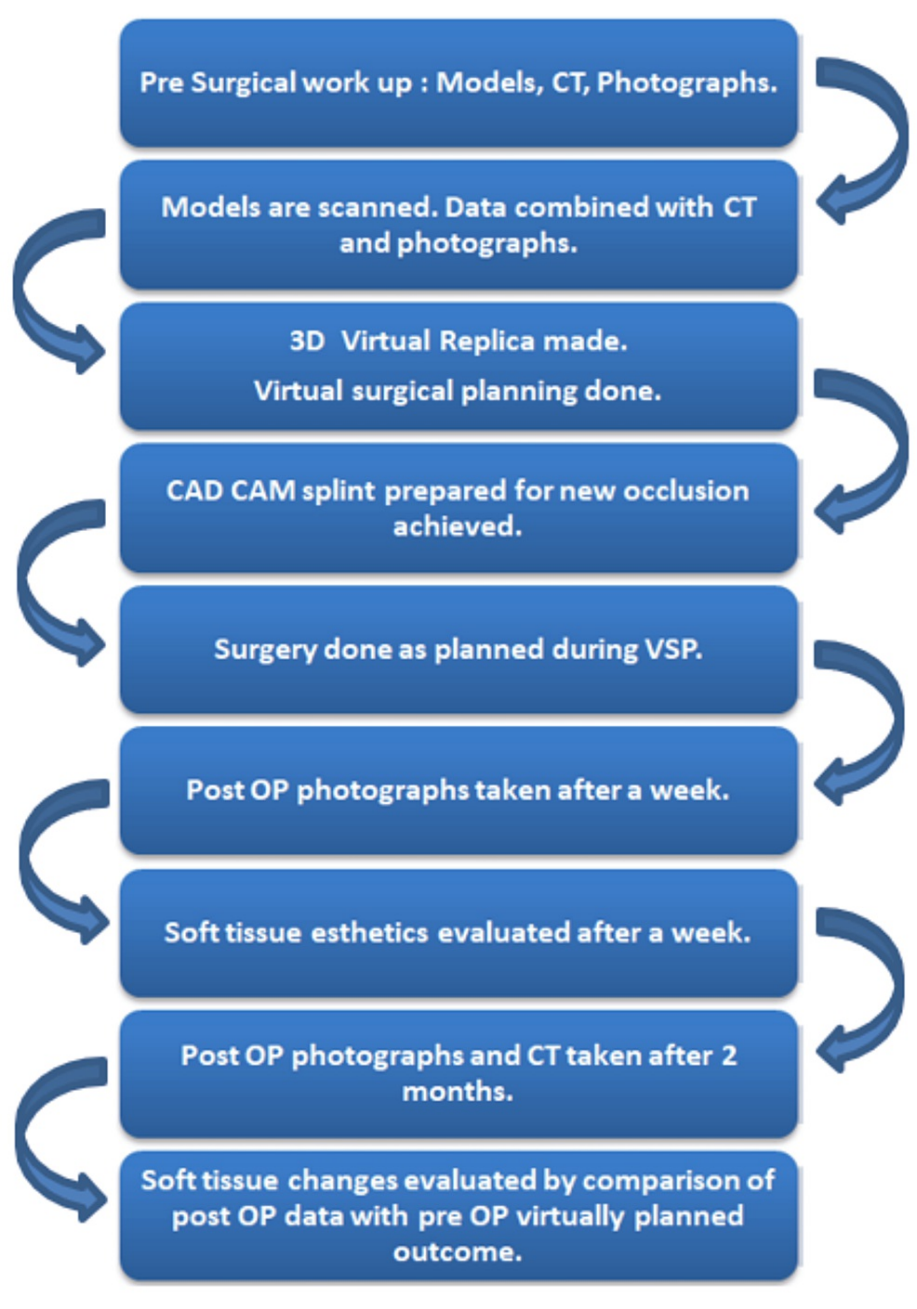
Flowchart of the Study Protocol CAD-CAM: Computer-aided design and computer-aided manufacturing; VSP: virtual surgical planning

**Figure 2:**
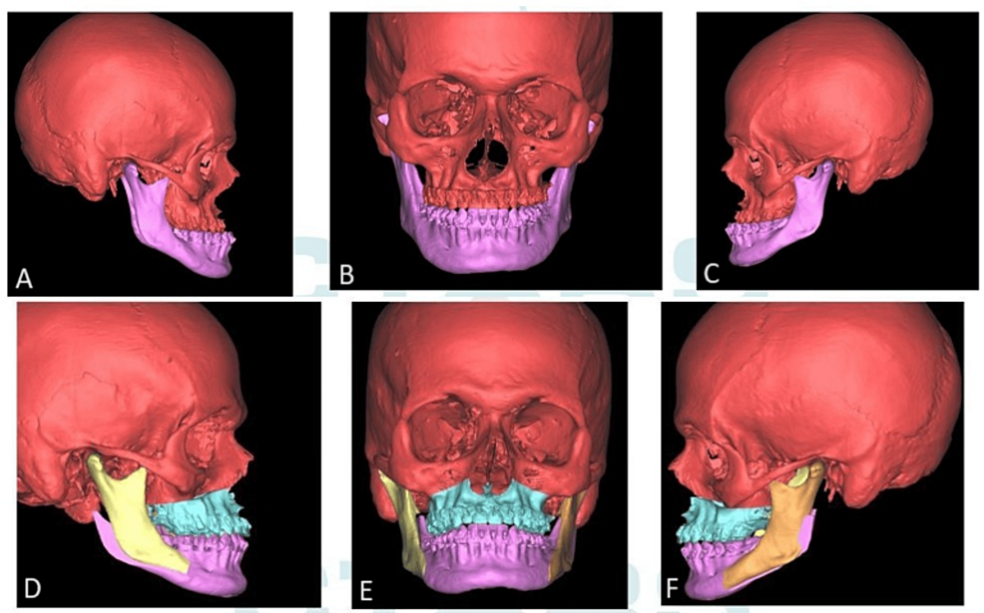
Virtual Surgical Planning on a 3D Replica A: Pre-operative right lateral view; B: Pre-operative frontal view; C: Pre-operative left lateral view; D: Post-operative right lateral view after maxillary advancement and mandibular setback; E: Post-operative frontal view after maxillary advancement and mandibular setback; F: Post-operative left lateral view after maxillary advancement and mandibular setback

**Figure 3:**
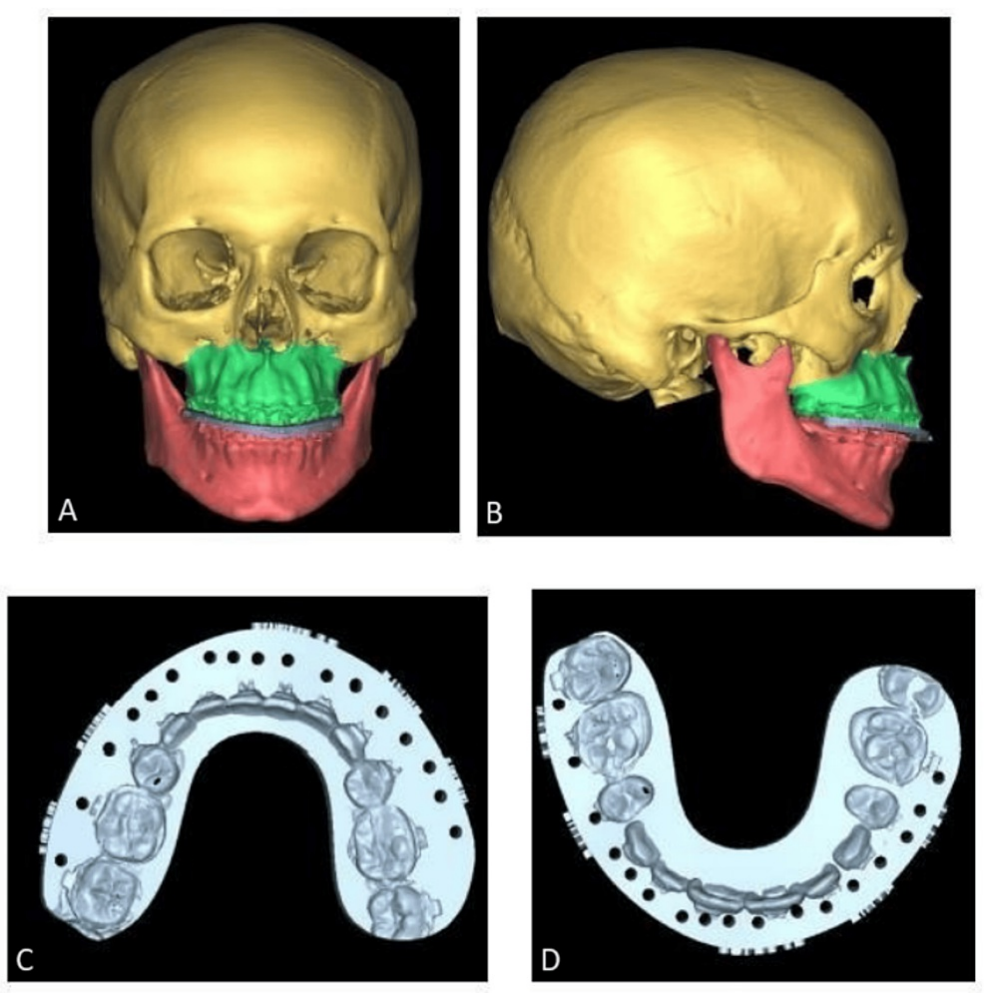
Designing of the CAD-CAM Splint A: Frontal view with the CAD-CAM splint; B: Lateral view with the CAD-CAM splint; C: Maxillary CAD-CAM splint; D: Mandibular CAD-CAM splint CAD-CAM: Computer-aided design and computer-aided manufacturing

Pre-surgical orthodontic treatment was done by the same team of specialists followed by pre-surgical clinical workup for all patients. A detailed case history was taken, and clinical examination was performed for all patients. Intra-oral and extra-oral photographs, OPG and lateral cephalograms were taken and cephalogram tracing was done. Dental impressions were taken, and models were prepared. A provisional surgical plan was devised for each patient. Models were scanned using a “SHINING 3D” dental scanner (SHINING 3D, Hangzhou, China) and data was converted and stored in DICOM format. Multi-slice spiral CT scan was taken at the same center where the same CT machine was used for all patients, to avoid magnification errors (128-slice CT scan with 0.6 mm slice thickness). CT data was combined with DICOM data using virtual surgical planning software and a 3D replica of the patient was arrived at. Virtual surgery was done on the replica to attain the desired maxilla-mandibular relationship using “MIMICS software by Materialize NV, Belgium” (Figure 2). Final splints were fabricated using “3D systems- Stereolithography (SLA) 3D Printers” (Figure 3). Surgery was performed as per the virtual plan, guided intra-operatively by the CAD-CAM printed splint (Figures 1-3).

Parameters evaluated

**Figure 4:**
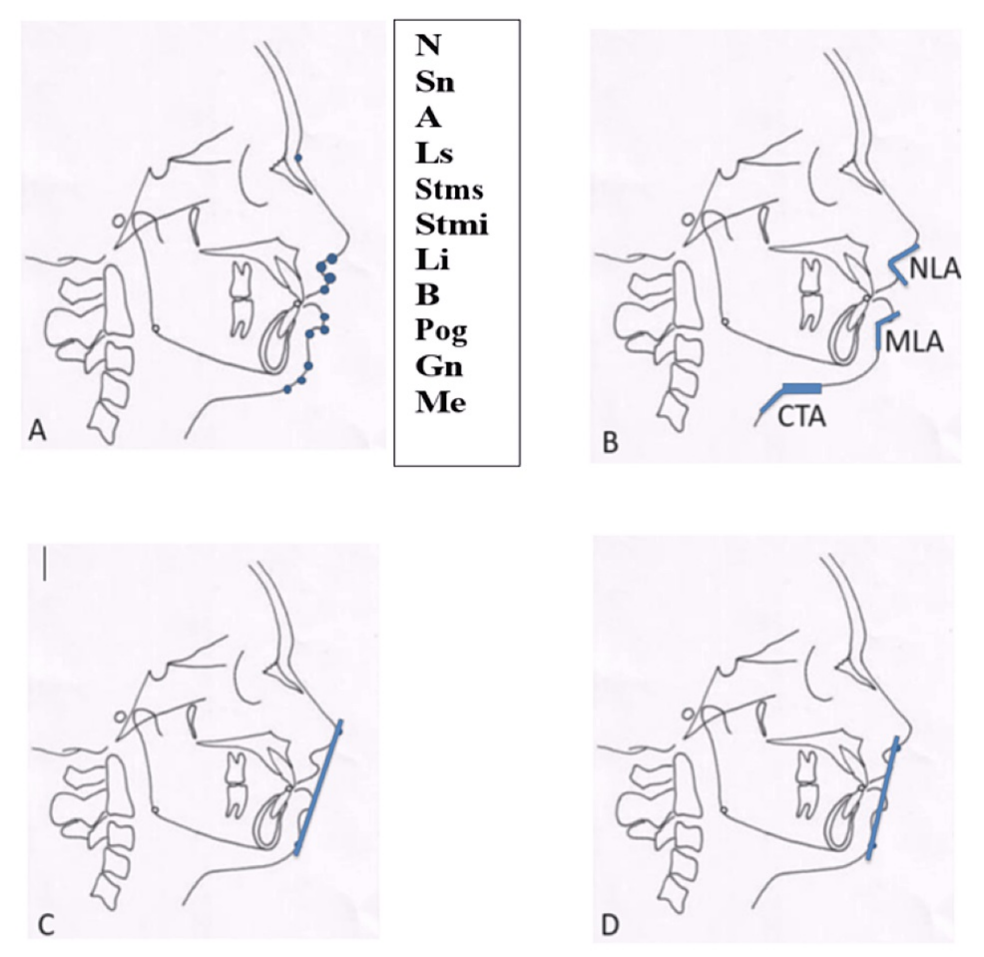
Landmarks Used A: N - Soft tissue nasion; Sn - Subnasale; A - Soft tissue point A; Ls- Labrale Superioris; Stms - Stomion Superioris; Stmi - Stomion Inferioris; Li - Labrale Inferioris; B - Soft tissue point B; Pog - Soft tissue Pogonion; Gn - Soft tissue Gnathion; Me - Soft tissue menton B: NLA - Naso-labial angle; MLA - Mento-labial angle; CTA - Chin throat angle C: Rickett's E line D: Steiner's S line

Pre-operative, virtual planning, and one-month post-operative soft tissue changes were evaluated by superimposing and comparing the positions of the selected soft tissue variable points in the virtual surgical planning software. The selected soft tissue points were marked on the Virtual Planning and post-operative 3D data and were superimposed to analyze the movements that have been achieved. The points were marked using artificial intelligence by the software itself. Linear and angular measurements were measured between various points. The values acquired pre-operatively, during virtual planning, and post-operatively were compared. Ricket’s E line and Steiner’s S line were also superimposed to analyze the postop outcome (Figure 4).

## Results

**Figure 5:**
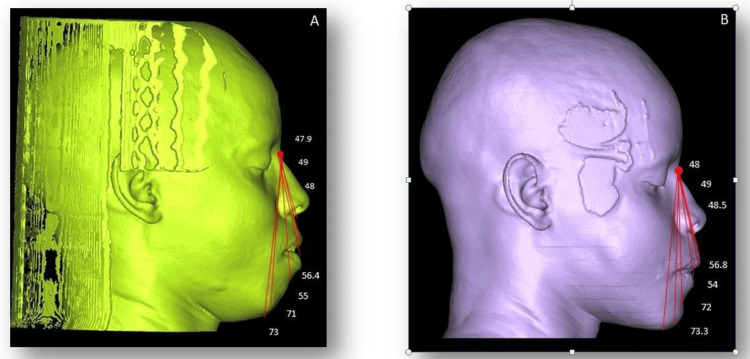
Measurements of the Virtual Outcome A: Pre-operative measurements; B: Post-operative measurements

Among the patients who reported for orthognathic surgery during the study period of two years, 12 patients were included in the study following the aforementioned inclusion and exclusion criteria. The age ranged from 18 to 30 years with a mean age of 23.5 years. Out of the 12 patients, 7 were males and 5 were females. Bi maxillary surgeries were performed on eight patients and four patients underwent only single jaw surgery. The accuracy of the VSP was assessed by comparing the position of the pre-decided soft tissue points of the virtual planning with one-month post-operative CT by superimposing on the software (Table 1). Linear distances were calculated keeping soft tissue nasion as the reference point (Table 2). Nasolabial angle, mento-labial angle, and chin-throat angular calculations were made (Table 3). Also, Rickett’s E line and Steiner’s S line were superimposed, and the distances were calculated (Table 4). All of these measurements were done for planned outcomes as well as post-operative outcomes to evaluate if the virtual planning has been effectively achieved intra-operatively. The mean discrepancy noticed after superimposition of soft tissue points was 0.92 with a standard deviation of 0.37. The mean discrepancy following the calculation of linear distances was -0.21 with a standard deviation of 0.44. The mean discrepancy following the comparison of angular measurements was -1.32 with a standard deviation of 0.83 (a negative value denoting the fact that the post-operative outcome was slightly behind the virtually planned outcome). The mean discrepancy after superimposition of the E line and S line was 2.54 with a standard deviation of 0.118 (Table 5). Thus, it is evident that although there were minor variations in comparison of virtual planning and post-operative outcomes in certain points, the discrepancies were negligible and carried no clinical relevance (Figure 5).

**Table 1:** Comparison of Values of the Virtual Outcome and Post-operative Outcome All measurements in millimeters.

Soft Tissue Points	P1	P2	P3	P4	P5	P6	P7	P8	P9	P10	P11	P12	Mean
Maxilla													
Subnasale	+1	0	+1	0	+3	+1	+1	0	0	+1	+1	0	0.75
Stomion Superiors	+2	+1.5	+1	+1	+2	+2	+2	+1	+1	+1	0	+1	1.29
Mandible													
Stomion Inferioris	0	0	0	+2	+1	0	0	+2	0	+1	+1	0	0.53
Pogonion	+1	+4	+2	0	+2	+1	+1	0	0	0	+3	+2	1.41
Gnathion	+2	+2	0	+1	0	0	0	0	0	+2	0	+2	0.75
Menton	0	0	0	+2	+1	+1	+1	+2	+2	+2	-1	0	0.83

**Table 2:** Difference in the Linear Distance N: Soft tissue Nasion; Sn: Subnasale; Stms: Stomion Superioris; A: Soft tissue point A; B: Soft tissue point B; Stmi: Stomion Inferioris; Pog: Soft tissue Pogonion; Gn: Soft tissue Gnathion.

Parameter	P1	P2	P3	P4	P5	P6	P7	P8	P9	P10	P11	P12	Mean
N-Sn	-0.2	-0.1	-0.4	-0.3	-0.4	0.5	0.9	-0.5	0	0	0.3	0.9	0.05
N-Stms	-0.8	0	1	-0.9	1	1.5	2	0	1	1	1	0.3	0.59
N-A	-0.4	0.5	0.2	-0.5	-1	0	1.2	-2	0	0	0	0	-0.16
N-B	-0.2	-0.4	1	-0.3	-1	1	-1	-2	-1	-1	-1	-1	-0.57
N-Stmi	1	1	1	0	-0.1	0.2	1	-0.2	-2	-2.1	-2	-1	-0.26
N-Pog	-2	-1	1.8	-3	0	-1	0.1	-1	1	1	1	-2	-0.42
N-Gn	-1	-0.3	0.3	-2	-1	-1	-1	-2	0	0	0	-1	-0.75

**Table 3:** Difference in Angular Measurements

Parameter	P1	P2	P3	P4	P5	P6	P7	P8	P9	P10	P11	P12	Mean
Naso Labial Angle	-2	0	-4.8	-3	-2	-2	-1	-3	-2	-2	-2	-2.8	-2.2
Mento Labial Angle	-2	0	3	-3	-0.5	0.5	3.5	-0.6	-3	-3	-3	2.7	-0.53
Chin Throat Angle	-3	-0.1	2	-4	-1	-1	-4	-2	-1	-1	-1	1.3	-1.23

**Table 4:** Superimposition of Planes All measurements in millimeters (mm). The positive value indicates that the postop outcome is ahead of the virtually planned outcome and the negative value indicates that the postop outcome is behind the virtually planned outcome. E line: Rickett’s E line; S line: Steiner’s S line

Parameter	P1	P2	P3	P4	P5	P6	P7	P8	P9	P10	P11	P12	Mean
E Line	3.4	5.5	2.3	3.3	1.7	1.7	2.6	1.6	1	2.3	1	4.9	2.46
S Line	3.7	5.8	2.5	3.6	1.5	1.5	2.9	1.4	1.3	1.5	1.3	4.6	2.63

**Table 5:** Mean and Standard Deviation Tabulation

Parameter	Mean	Median	Mode	Standard Deviation
Superimposition of Soft Tissue Points	0.92	0.97	0.75	0.37
Linear Measurements	-0.21	-0.57	-	0.44
Angular Measurements	-1.32	-0.53	-	0.83
Superimposition of Planes	2.54	2.54	-	0.118

## Discussion

This study aims to assess the accuracy achieved by using VSP for orthognathic maxillo-mandibular movements by comparing the changes in soft tissues in the VSP simulation and post-operative 3D CT reconstruction. The 3D planning was done with the help of “MIMICS software by Materialize NV, Belgium” which helped in diagnosis, virtual surgical movements, soft tissue prediction, and manufacturing of CAD CAM surgical splints that were used intra-operatively to obtain the predicted results.

The splint fabrication is the most vital step in VSP as it guides the surgeons in achieving the desired jaw movements [5]. They play a significant role in bimaxillary surgeries as they make the complex multi-planar movements of both jaws easier and more predictable. In a bimaxillary surgery, we use two splints, an intermediate and a final splint which help in guiding the movements of the two jaws precisely into desired positions [6].

The conventional planning usually done by performing mock surgery on dental models, manual articulation and hand-made splints are not only cumbersome and time-consuming but also are prone to human error [7]. 

It is also important to note that the models only represent the teeth and not the surrounding bone in which the actual movement occurs. In VSP, the bone and surrounding vital structures are simulated by the software, and precise movements of osteotomized segments can be done by the surgeon to evaluate the anticipated difficulties that may arise intra-operatively [8].

Fabrication of splints by hand is extremely difficult as following the mock surgery done on dental models by performing osteotomy cuts on them, and then moving the models to the desired position, they have to be stabilized in the newly achieved position after which the splints have to be manufactured manually. The acrylic material made to fabricate these splints is difficult to manipulate and mold. They are at risk of warpage due to temperature changes and external factors prior to usage intra-operatively.

In VSP, the 3D printed splints are made of resin materials which have a better dimensional stability compared to the conventional acrylic splints and adapt well to the dentition intraoperatively as no distortion of the material is expected. It is also of note that the condyle fossa relationship is kept constantly in centric relation throughout the planning process which is imperative to avoid post-operative complications.

An important factor worth considering in VSP and fabrication of cutting guides is the time consumed by the residents in the entire process, studies have shown that the 3D CT planning has drastically reduced the total time spent by the residents for the planning and splint fabrication, and there was a reduction by 45% in the total time consumed as shown in the study by Wrzosek et al. [9].

A major advantage associated with virtual planning is the elimination of all physical elements involved. As the process is completely digital, data can be transferred and stored seamlessly. The simulation could be used to advise the patient regarding the final outcome that could be expected [9]. It is usually assumed that soft tissues always follow the hard tissues following osteotomy, but this may not be entirely true. The software simulates not just hard tissue movements but also the soft tissue changes that occur following such movements, thus eliciting a complete 3D reconstruction. The 3D prediction of the surgical outcome is an important advantage in 3D CT planning [10].

Considering all the drawbacks associated with traditional pre-surgical planning it is wise to conclude that virtual 3D CT planning may play a vital role in achieving precise post-operative results in the upcoming years. Thus, it is important to analyze the accuracy that could be achieved by VSP in order to recommend it for regular clinical and surgical practice.

Quereshv et al. studied the results achieved by virtual planning statistically and arrived at a conclusion stating that no “statistically significant” difference was present between the planned and the achieved orthognathic movements. A mean difference of 2 mm was seen which was not statistically significant [11].

Lin et al. published a literature review in 2018 on “3D printing in orthognathic surgery” and presented data regarding the use and benefits of the technology. They arrived at the opinion that 3D Printing is beneficial in providing satisfactory aesthetic and functional outcomes by accurate transfer of the planned data to an operating room [12].

Though soft tissue changes can be predicted with the 3D planning software, no studies are available to assess the reliability of these predictions [13]. Our study has aimed to evaluate if a virtually planned outcome has been achieved post-operatively by incorporating 3D CT orthognathic planning by comparing the soft tissue changes seen in the planned outcome with the post-operative outcome. The mean discrepancy seen in the former was -0.21 ± 0.44 and in the latter was -1.32 ± 0.83 (negative denoting the fact that the post-operative outcome was slightly behind the virtually planned outcome. This could be due to slight intra-operative bone loss during osteotomy when compared to virtual osteotomy). These variations could be attributed to relapse, unpredictable soft tissue movement following bony movements, minute changes from the virtual plan as decided by surgeons, intra-operatively, or due to other external factors not under our direct control. These variations are minute and not clinically significant. Thus it can be concluded that VSP is an effective, predictable, and accurate method that can be reproduced in most patients undergoing orthognathic surgery.

Although several studies have compared the predictability of VSP, most of them have used nonstandard complicated reference points and lines for evaluation of results. In this study, as we have used standardized cephalometric landmarks, the same can be adapted by other surgeons and practitioners in their clinical practice [7].

Further advances in technology have now incorporated tools like patient-specific implants, surgical cutting guides, navigation technology, artificial intelligence-assisted surgery, robotic surgeries, etc. Such tools can revolutionize the field of Orthognathic Surgery as they minimize the possibilities of human error and reduce the intra-operative time to a bare minimum. Using such tools, precise osteotomy cuts can be achieved leading to perfectly desired jaw movements. With the advent of these tools, splintless surgery is now a possibility [14].

In our opinion, the only limitation of 3D CT planning is the high expenditure involved in the procedure. This may be due to the high technical knowledge that is required, the cost involved, and also due to lack of awareness. The need for a CT scan may also increase the overall cost involved along with the debate of radiation exposure to the patient. There are no studies that have compared the conventional and 3D printed splints, making it difficult to conclude statistically if the 3D printed splints give better results compared to the conventional splints. More studies in this domain would make 3D CT VSP a more reliable tool in Orthognathic Surgery. A larger sample size would also provide more credibility to the study.

## Conclusions

3D CT VSP is a reliable tool to achieve predictable and reliable post-operative results in orthognathic surgical cases. Proper surgical planning, accurate diagnosis, correct sequencing, and good surgical technique are needed to make 3D CT planning successful. Newer technology such as VSP, CAD CAM splints, custom surgical guides, and navigation makes the future of oral and maxillofacial surgery both challenging and exciting for the surgeon while being more predictable for the patient with better post-operative results.
